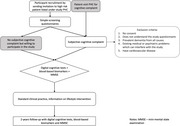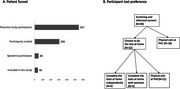# The PREDEM primary care study ‐ targeted screening of people at risk of developing Alzheimer´s disease dementia in primary care ‐ study design and feasibility assessment

**DOI:** 10.1002/alz70861_108761

**Published:** 2025-12-23

**Authors:** Sandar Aye, Signe Åhrberg, Sara Wallén, Wobbie van den Hurk, Ibrahim Alshamani, Lena Lund, Axel C Carlsson, Linus Jönsson, Lars O Tjernberg, Sophia Schedin Weiss

**Affiliations:** ^1^ Karolinska Institutet, Solna, Region Stockholm Sweden; ^2^ Academic primary health care center, Stockholm, Region Stockholm Sweden; ^3^ Mindmore, Stockholm, Region Stockholm Sweden

## Abstract

**Background:**

Early and accurate diagnosis of Alzheimer’s disease (AD) is crucial for early intervention using disease‐modifying therapies. Primary healthcare centers (PHCs) play a key role in recognizing individuals at‐risk, as they are the first contact for memory complaints. Targeted screening for those with cognitive symptoms and dementia risk factors could be a realistic strategy to increase detection of early AD. For this purpose, blood‐based biomarkers (BBMs) combined with digital cognitive tests would be highly informative.

**Method:**

This longitudinal prospective study aims to establish an efficient workflow to identify individuals at risk for AD in a primary care setting. Participants are recruited from PHCs in the Stockholm region and are subjected to screening questions, a digital cognitive test battery and BBM analysis. The cognitive tests and BBM analysis will be repeated after two years. Participants are stratified into three profiles: a) ≥65 years b) ≥65 years with hypertension, and c) ≥65 years with type 2 diabetes with or without hypertension. 400 participants per stratum will be recruited.

**Result:**

A pilot study has been conducted to assess the feasibility of the study design. 547 potential participants were identified from electronic medical records. After manual review, 306 were invited to participate, and 40 (13%), all of which experienced subjective cognitive complaints (SCD), were included. Of these, 55% preferred to do digital cognitive testing at home, but only 25% completed it, and only 15% completed the tests without assistance. The mean age was 72 years, 58% were female, and nearly half had a family history of dementia. 82% were not worried about memory symptoms, and 62% reported no worsening over time.

**Conclusion:**

The main study is ongoing. The pilot study shows feasibility in recruiting patients with dementia risk factors and using digital cognitive tests. The limited technical capability among some participants showed a need to establish digital test equipment at the PHCs. The high attrition is due to the low proportion of SCD cases and gives an estimate on the number of participants that can be expected in an AD screening program. Results will inform the clinical applicability and economic benefits of targeted screening.